# How Might the Relation of the Development of Hand Preferences to the Development of Cognitive Functions be Examined During Infancy: A Sketch?

**DOI:** 10.3389/fnins.2017.00739

**Published:** 2018-01-08

**Authors:** George F. Michel

**Affiliations:** Department of Psychology, University of North Carolina at Greensboro, Greensboro, NC, United States

**Keywords:** hand preference, infants, embodied cognition, cognitive development

## Abstract

Investigations of the relation of the development of hand preferences to the development of other sensorimotor and cognitive abilities are plagued by confusing and contradictory results. In part, the confusion derives from the failure to create accurate, appropriate, and reliable descriptions of the development of hand preferences and the cognitive or sensorimotor ability of interest. This paper sketches an ideal longitudinal study (from birth through 5 years) with a large sample size that should provide reliable evidence for the understanding of the relation of hand preferences to cognitive development. Since hand preference differences would affect the way infants engage in manual actions with objects and these differences would likely affect how they come to comprehend object relations, differences in the development of cognition across handedness groups would be a good test of certain forms of embodiment theory.

## Introduction

For more than four decades, I have been investigating the development of hand-use preferences during infancy. That work has identified a potential origin for the hand preference in the newborn postural asymmetry (which, in turn, may reflect intrauterine postural asymmetries) that creates a head orientation preference (HOP, Michel, 1981). The overwhelming majority of infants (68%) exhibit a reliable HOP to keep their head oriented toward their right side when supine (or seated at a 45° angle); whereas, a minority (12–14%) exhibit a reliable HOP to keep their head to their left side. The remaining 18–20% exhibit no reliable HOP.

The left or right direction of neonatal HOP predicted initial hand-use preference for contact with, and the obtaining of objects (Michel and Harkins, 1986). We proposed that this prediction was the result of the influence of HOP on arm and hand movements and the visual, tactile, kinesthetic, and proprioceptive (and perhaps even CNS corollary discharges) feedback such laterally asymmetric movements created. Such sensory feedback likely established sensorimotor circuits in the nervous system that ensured more precise sensory control of the movements of the “face-side” arm and hand resulting in an advantage for the face-side hand for object acquisition and manipulation. Moreover, we proposed that such circuits could be used as the foundation for the establishment of other sensorimotor neural circuits that would contribute to the embodiment of cognitive functions (Michel et al., 2013a, 2016).

To assess infant handedness from 5 to 14 months, my colleagues and I created an assessment procedure that was both reliable and validated on a block play session (Michel et al., 1985). In this assessment, we identified three types of manual skills when manipulating toys/objects for which hand-use preferences could be identified. These three skills were the acquisition of objects, the unimanual manipulation of objects, and the role-differentiated bimanual manipulation (RDBM) of objects. Moreover, acquisition hand preferences seemed to precede unimanual manipulation preferences and both preceded RDBM preferences during development. Indeed, we proposed that an apparent decline in the acquisition preference after 12 months was likely the consequence of acquisition skills being employed in the service of RDBM skills. That is, as the sensorimotor circuits for acquiring objects became more efficient, they could be “attached” to (or associated with) circuits for RDBM. Thus, the non-preferred hand could be employed to obtain the object so that the preferred hand could immediately initiate RDBM without the need to transfer the object into the non-preferred hand. Subsequent research demonstrated that the hand preference for acquisition predicted the later developing hand preference for unimanual manipulation (Campbell et al., 2015a) and the even later developing hand preference for RDBM (Nelson et al., 2013a; Babik and Michel, 2016).

Therefore, I proposed a cascade hypothesis for the development of hand preferences during infancy (Michel et al., 2013b). Initially, a head orientation preference facilitates the development of a preferred hand for visually-guided object acquisition. The preference for acquisitions concatenates into a preference for unimanual manipulation because the acquiring hand can do many more manipulations (shaking, hammering, squeezing, to-mouth actions, even transfer actions to the other hand) before the non-preferred hand obtains the object. Thus, sensorimotor circuits of acquisition adhere to circuits for acting on the object. Since RDBM requires circuits for one hand's manual exploration of the object while the other hand supports such exploration, we proposed that the acquisition/unimanual object manipulation hand preference would concatenate into a hand preference for RDBM. Several studies provide indirect support for this (Nelson et al., 2013a; Babik and Michel, 2016) but we are currently examining a very large sample of infants to assess the path connection acquisition, unimanual, and RDBM handedness during the period from 6 to 14 months of age.

Additionally, we discovered that the infant's hand preference is affected by the mother's hand preference (Michel, 1992) likely because of the way mothers unintentionally shape their infant's hand use during their dyadic play (Mundale, 1992). As have others, we noted that left handed mothers do not use their left hand in a mirror image of the frequency and character of the use that right-handed mothers make with their right hand. Indeed, whereas right-handed mothers can be strikingly dominant in their use of their right hand, left-handed mothers merely show a moderate bias in their left-hand use. This means that infants who are developing a left-hand preference (based upon their head orientation preference) who have right-handed mothers (a likely occurrence because right-handedness predominates in the population) will have their left-hand preference unintentionally weaken by their mother's hand use during dyadic play. Similarly, infants developing a right-hand preference will have their right-hand use strengthened by their right-handed mothers.

In contrast, infants who are developing a right-hand preference (most infants) will very likely not have left-handed mothers, but if they do, their dyadic play will only mildly affect the infant's right preference because left-handed mothers are not as strongly handed as are right-handed mothers. Of course, infants with left-handed mothers who are developing a left-hand preference because of their left head orientation preference will have their left-hand use strengthened by dyadic play with their left-handed mothers. However, their left hand preference will not be strengthened as much as infants who are developing a right preference with their right-handed mothers. Thus, parental interaction (or indeed, societal proscriptions) can affect the development of the hand preference during and after infancy.

Although some of our studies may have been underpowered and may have generated controversy (but not failures to replicate), we felt comfortable in our assessment of the development of infant hand preferences as a cascading process. Therefore, we began to examine the relation of such development to the development of other cognitive functions. We (Kotwica et al., 2008) observed that a hand preference for acquisition facilitated the development of object storage skills (an ability considered to reflect early symbolic knowledge, Bruner, 1973). Also, an acquisition hand preference predicted advanced language skills a year later at 2 years of age (Nelson et al., 2013b). Consistency of a hand preference across infancy (6–14 months) and toddlerhood (18–24 months) predicted advances in language skills at 3 years of age (Nelson et al., 2017). An infant hand preference predicted advances in infant and toddler object construction skills (i.e., stacking blocks) that is thought to both reflect and contribute to the development of spatial knowledge (Marcinowski et al., 2016).

These predictive relations are only suggestive of an influence of infant hand preference development on cognitive development. Moreover, there have been proposals that infant hand preferences are tied to developing postural control and hence are not consistent during early development (Corbetta and Thelen, 2002; but see Babik et al., 2014; Campbell et al., 2017). Also, infant preferences have been argued to be unrelated to later childhood and adult hand preferences (Dubois et al., 2009). To further confuse the issue, there is some evidence (Esseily et al., 2011, but see Cochet et al., 2011) that infant hand preferences when engaged with objects is not related to their hand preference for the gestural communication skill of pointing. Unfortunately, many of these contradictory studies come from underpowered studies with statistically indefensible classifications of hand preference and too few instances of data collection needed to identify longitudinal trajectories in either hand preference or the cognitive/communication skill examined (Campbell et al., 2015b). This has prompted me to sketch the ideal design for studies that would effectively reveal whether there is any relation between infant/toddler hand preferences and the development of cognitive abilities such as language skills, gestural communication, spatial knowledge, problem-solving, and tool construction and use.

The remainder of this paper will sketch out this design with full recognition that no agency would fund it and that it would take at least two decades to complete (unless multiple labs undertook simultaneous identical investigations to permit achievement of the needed sample size). The power of this design derives from its ability to address most of fundamental problems associated with the investigation of the relation of the development of infant hand preferences to cognitive development.

## The ideal design (A sketch)

The examination of the developmental relation between any two psychological functions or skills requires two essentials: (1) the investigator's ability to adequately, appropriately, and reliably (AAR) describe hand preference and the cognitive ability of interest across a developmental period; (2) the investigator's ability to collect longitudinal data that can identify developmental trends in the expression of each function or skill. For the most part, the investigation of the relation of hand preferences to any cognitive function or skill (e.g., language, problem-solving, spatial understanding, numerical skills, tool-use, and artifact construction skills, etc.) has been conducted in a rather *ad-hoc* manner.

For example, when examining the question of whether an infant's hand preference is related to either development of gestural communication or a hand preference in gestural communication, a study may be designed which examines infant hand preference on some task (or using some assessment technique) at a particular age (or across a few ages, seemingly selected by convenience rather than by interest in identifying continuity or change in the hand preference during this developmental period) and the results are compared to the data collected from the same infants using an some assessment of gestural communication (pointing) or of a hand preference for gestural communication. Similar designs have explored infant hand preference and language (production and reception), tool-use, spatial skills, etc. A relation may or may not be observed but little is offered about how these functions (development of a hand-preference and development of the cognitive function) should identified and specified and how best their developmental relation should be examined. Both of these functions require AAR descriptions across longitudinal designs.

## Adequate, appropriate, and reliable (AAR) descriptions across development

For investigating the relation between hand preference and cognitive functions, this question bifurcates into: Are there AAR developmental descriptions of infant hand-preferences? Are there AAR developmental descriptions of the cognitive functions of interest (e.g., gestural communication, tool-use, artifact construction, spatial language skills, etc.)? Since many of the design characteristics that are essential for AAR descriptions of hand preference development may be applied to the developmental descriptions of other cognitive and sensorimotor skill, this paper will simply focus on the study of hand-use preferences with occasional references to the development of other abilities.

What would be an AAR for assessment of infant hand preferences? No assessment of an infant's ability could capture all the variability both within and among individuals during the development of any cognitive skill. It has long been noted (Annett, 1964) that adult handedness is continuously distributed (as assessed by either performance measures or questionnaire) across individuals despite our common tendency to classify people into discrete categories. A continuous variable with a right skew can only be categorize into classes using statistically defensible criteria. Self-assignment would not be defensible because performance differences and/or answers to questions may be strikingly different between individuals using the same self-assigned class. Moreover, the individual has a lifetime to establish, perfect and manifest a hand preference; any assessment can only reveal snapshots of that process.

We have shown that the preference from birth to 2 years of age must be examined across several manual actions [arm-swiping at visual objects, manually contacting, and acquiring seen objects, manipulating objects with one hand (banging, shaking, tapping, fingering, etc.)], manipulating objects with both hands but in a manner of role-differentiated bimanual manipulation (RDBM). RDBM requires that one hand facilitates the actions the other hand. Thus, the supportive hand enables exploration of the features of the object by the preferred hand or the supportive hand steadies the object while the preferred hand stacks an object on it or uses a tool to alter the object (chipping stone-tools, tying arrowhead to spear shaft). Note that most adult hand preferences are manifest during RDBM actions.

Hand preferences for these infant manual actions become manifest at different ages (swiping from 2 to 5 months, acquisition from 5 to 14 months, unimanual manipulation from 9 to 12 months, RDBM from 12 months to?). Thus, an appropriate assessment during infancy would have to include separate assessments of hand preferences for each of these actions because these are characteristic examples of the types of manual actions in which a hand preference may be exhibited. The assessment would be adequate if it included enough instances of each type of action to estimate the probability that any preference exhibited in the action would be unlikely to occur by chance. The assessment task should be reliable in that re-assessment within the same age does not alter the infant's apparent preference. Of course, the preference is likely to show a somewhat continuous character in the frequency of each hand's use across assessments and individuals, but it should be possible to statistically identify potential preferences that fit three general categories of right, left, and no statistically reliable preference. With a large enough sample size, the sample may be divided further (statistically) to reveal additional sub-groupings (those developing a preference earlier than others) within these three general categories.

Therefore, AAR assessment of infant handedness would require a large sample with testing that would have to be conducted across a 2+ decades. Evidence from published studies suggest that a sample of 400 (in rolling cohorts of about 40 longitudinally examined across their first 3 years and tested once again at 5 years of age) would provide some 60+ infants who are likely to exhibit a leftward HOP during their first 10 neonatal weeks, most of whom would likely develop a later left-hand preference and about 80+ would have no reliable preference for either HOP or hand-use. These sample sizes would permit reliable estimation of the relation of a left or right preference relative to no preference for the development of any cognitive ability.

More importantly, this sample would permit experimental manipulation of manual feedback influences on the asymmetry of neural monitoring of each hand created by the HOP. For example, using a variant of Needham's “sticky mittens” procedure, each of the three HOP groups (identified by three assessments of the HOP at 3, 6, and 9 weeks postpartum) would be divided into three groups: those who would have no mittens experience but be exposed to the testing procedures, a group who would have mittens on both hands, and a group who would have mittens on their “face-side” hand as determined by the HOP. For those infants without a HOP, a third (about 25–30 infants) would be randomly assigned a mitten hand. The mittens are worn for a week starting at 12 weeks' post-partum and their swiping at objects and their evoked potentials to vibrational stimulation of their fingers on their right and left hands would be tested before and after the week of sticky mitten experience.

If the asymmetrical feedback from HOP has an influence on neural circuits associated with control of the arms/hands, then before the sticky mitten manipulation, both the right and left HOP infants should exhibit greater evoked potentials (EPs) to stimulation of their face-side hand than their skull-side hand and there should be no differences among the infants without a HOP. After sticky mitten experience, the asymmetry in the EPs should be greater for those infants with a HOP and there should be an asymmetry apparent for those infants without an HOP but who had the asymmetrical sticky mitten experience. If there is no asymmetry of EPs associated with HOP or sticky mitten experience, then it is possible that the sticky mitten experience does not create the feedback common for the construction of the asymmetric circuits in the brain or it is possible that the asymmetric monitoring of the hands created by the HOP (and enhanced by the sticky mitten experience) does not help sculpt neural circuits involved in hand control. These results would provide an answer to the basic question of whether the infant's neural development is, in part, shaped by its own self-generated experiences.

The assessment of HOP at only three ages does not permit description of the developmental trajectory of HOP. However, the assessment or hand preferences (for acquisition, unimanual, RDBM actions) every 2 months from 5 to 25 months (11 assessment periods) permits replication of our published developmental trajectories for hand preferences for acquisition and unimanual manipulation. Also, it permits identification of the developmental trajectory of hand preference for RDBM from 11 to 25 months (8 assessment periods). This latter data enables us to connect the first year RDBM hand preferences to RDBM hand preferences in the second year and estimate their developmental trajectories. Moreover, collection of data on construction skills (e.g., stacking), tool-use, and pointing conditions from 11 to 25 months would provide sufficient data to identify trajectories in the development of these abilities as well as hand preferences within each.

Of course, identifying hand preferences in these sensorimotor skills requires enough instances of the manual actions manifest in each to reliably eliminate chance in any apparent hand preference. Thus, for a hand preference for pointing, there would need to be at least 15 and better 20 instances of unimanual pointing for each age period. These same infants can be tested bimonthly from 23 to 35 months of age (7 assessment periods) on various specific language skills (e.g., the use of spatial prepositions) and a common preschool hand preference task (Scharoun and Bryden, 2014) could be administered at 33 and 35 months of age. The latter could reveal how well measures of hand preference in early infancy predict later hand preferences and how these later preferences relate to concurrent language ability.

For a more conventional measures of language development, the MacArthur Communicative Development Inventory (MCDI) could be collected around the ages for hand preference assessment. The MCDI provides a caretaker assessment of language development with an infant component useful from 8 to 16 months postpartum and a toddler component useful from 16 to 30 months. In contrast, another conventional language assessment task, the Preschool Language Scale (PLS-5), is an experimenter administered assessment that can be conducted in a lab or home setting and could provide standardized measures of the child's language skills at two and three years of age. Both these assessments could be compared to previous and concurrent measures of hand preference.

It is easy to extrapolate from the assessment of language skills to the assessment of any other cognitive skill for this age range. So, this design Sketch has some general developmental utility. By itself, it can elucidate the relation of hand preference development with object manipulation to the hand preference exhibited during gestural communication. The design could reveal also whether the assessment of infant hand preferences relate to hand preferences exhibited in construction actions and tool-use as well as developmental advances in these skills relative to infant hand preferences. The design permits identification of whether infant and toddler hand preferences relate to common assessments of preschool hand preference (see Tables 1A,B for a sketch of some of the tasks and hypotheses that could be tested with this longitudinal assessment design. Note that it would be easy to include additional cognitive, social and emotional tasks at additional ages to assess the effects of handedness on the development of these abilities and certain aspects of embodiment theory).

**Table 1A:** Tasks and hypotheses tested during infancy (*n* = 400).

**Age**	**Tasks**	**Hypotheses tested**
3, 6, 9 Weeks Postpartum	Assessing Head Orientation Preference (HOP)	Does HOP affect lateral asymmetries in self-touching and arm and hand movements?
12, 15, 18 Weeks	1 week of sticky mitten experience at 12 weeks (4 Groups: Face side hand only, both hands, non-sticky mittens)	Is there greater swiping at objects with face side hand and more so after wearing sticky mitten?
	ERPs from contralateral vibration of fingers of left and right hands	Does face side hand stimulation provokes greater ERP than skull side hand; Does hand that wore sticky mitten provokes greater ERP than face side hand alone; Does earlier HOP should predict hand used most frequently when swiping at objects at 18 weeks?
10, 12, 14, 16, 18, 20 Months	Role Differentiated Bimanual Manipulation (RDBM	What is the pattern of development of hand preference for RDBM; Does earlier HOP and swiping hand preference predict hand preference for RDBM?
	Pointing	Does RDBM hand preference predict developmental trajectory of pointing; a hand preference for pointing?
	Tool-use	Does RDBM hand preference predict developmental trajectory of tool-use; a hand preference for tool-use?
	Object Construction	Does RDBM hand preference predict developmental trajectory of construction skills; a hand preference for construction?
	MacArthur-Bates Communicative Development Inventory (MCDI)	Does RDBM hand preference predict developmental trajectory of language skills?

**Table 1B:** Tasks and hypotheses tested during preschool years (*n* = 400).

**Age**	**Tasks**	**Hypotheses tested**
24, 30, 36, 48 Months	Preschool Language Scale-−5th Edition (PLS-5)	Does infant RDBM hand preference predict earlier development of language skills?
	Scharoun and Bryden Handedness Assessment	Does infant RDBM hand preference predict development of child hand preference assessment; Does child hand preference predict concurrent language skill?
	RDBM Handedness Assessment	Does RDBM hand preference remain stable during early childhood; Does child RDBM hand preference predict concurrent language skill; what is the relation between the two hand preference assessments?
	Theory of Mind (ToM) Tasks	Examine relation of handedness to ToM; Do infant hand preferences predict differences in ToM development; Do current hand preferences predict differences in ToM development?
60 Months	School Readiness Test-−4th Edition (SRT-4)	Do infant and child assessments of hand preference predict differences in SRT performance?
	Bender Gestalt Copy Designs Test (BGT)	Do infant and child assessments of hand preference predict differences in copy design performance?
	Scharoun and Bryden Handedness Assessment	Do child hand preferences predict hand preferences at 5 years?
	RDBM Handedness Assessment	Do infant and/or child hand preferences for RDBM predict hand preferences at 5 years?
	Theory of Mind (ToM) Tasks	Examine relation of ToM to SRT and BGT

Moreover, if these children can be examined again at 5 years of age, they could be given school readiness tests, children's handedness assessments, and some common cognitive tasks that could be related to their concurrent and earlier collected hand preference and language data. Two+ decades of such intensive data collection and analysis could provide the most AAR data on the relation of early hand preference development to various language and other cognitive skills. Moreover, it would set the investigation of early psychological development on a path that requires programmatic, longitudinal, large sample research designs that could only improve our understanding of psychological development.

## Conclusion

There is growing theoretical interest in, and experimental support for, various forms of embodiment theory of cognitive development. Clearly, a hand-use preference is the most common and distinctive source for the formation of differences in embodied sensorimotor actions. Hand preference differences during infancy and toddlerhood would matter in profound ways for things like object exploration, artifact construction, and tool use. How one holds the object and what infants see of the object as they engage in manual actions is going to differ. The information infants collect and possibly how/if they engage with another person around these activities could be different depending on their hand preference. Therefore, differences in cognition across handedness groups is a good test of certain forms of embodiment theory (Casasanto and Henetz, 2011).

Since there is much evidence that the development of left-hand preferences is not the mirror image of the development of a right-hand preference, sample sizes need to be very large to have the power to identify whether a hand preference or a specific (left or right) preference directly relates to the development of any cognitive ability. Although certain forms of embodiment theory predict that a hand preference ought to relate to many cognitive abilities, the effects of the difference in the development of a left verses a right preference must be examined because they, too, ought to create differences in cognitive ability. I propose that investment in the collection via the design sketched here would provide greater payoff than continued investment in the more *ad-hoc* projects.

## Ethics statement

This study was carried out in accordance with the recommendations of the Institutional Review Board for the protection of human subjects of the University of North Carolina Greensboro with written informed consent from all subjects. All subjects gave written informed consent in accordance with the Declaration of Helsinki. The protocol was approved by the UNCG IRB.

## Author contributions

The author confirms being the sole contributor of this work and approved it for publication.

### Conflict of interest statement

The author declares that the research was conducted in the absence of any commercial or financial relationships that could be construed as a potential conflict of interest.
